# Supplementary material for: Performance comparison of QuantiFERON-TB Gold In-Tube and QuantiFERON-TB Gold Plus in detecting Mycobacterium tuberculosis infection among HIV patients in China
Source: PeerJ. 2025 Mar 31;13:e19195. doi: 10.7717/peerj.19195 (PMC11967438; doi:10.7717/peerj.19195)
Supplement: Supplemental Information 1 [file peerj-13-19195-s001.docx]

STROBE Statement—checklist of items that should be included in reports of observational studies

|  | Item No. | Recommendation | Page  No. | Relevant text from manuscript |
| --- | --- | --- | --- | --- |
| **Title and abstract** | 1 | (*a*) Indicate the study’s design with a commonly used term in the title or the abstract | 1,2 | No direct comparative study assessing QuantiFERON-TB Gold In-Tube (QFT-GIT) and QuantiFERON-TB Gold Plus (QFT-Plus) for *Mycobacterium* tuberculosis infection among (Persons living with HIV) PLHIV in China has been conducted. |
|  |  | (*b*) Provide in the abstract an informative and balanced summary of what was done and what was found | 1,2 | No direct comparative study assessing QuantiFERON-TB Gold In-Tube (QFT-GIT) and QuantiFERON-TB Gold Plus (QFT-Plus) for *Mycobacterium* tuberculosis infection among (Persons living with HIV) PLHIV in China has been conducted. |
| Introduction | | | |  |
| Background/rationale | 2 | Explain the scientific background and rationale for the investigation being reported | 5 | Nevertheless, as far as our understanding extends, no direct comparative study assessing QFT-GIT and QFT-Plus for diagnosing *Mycobacterium* tuberculosis infection among PLHIV in China has been conducted. |
| Objectives | 3 | State specific objectives, including any prespecified hypotheses | 5 | Therefore, we conducted this cross-sectional study to compare the performance of QFT-GIT and QFT Plus for diagnosing *Mycobacterium* tuberculosis infection among PLHIV in China. Moreover, we compared quantitative data of IFN-γ production of QFT-GIT after preventive treatment. |
| Methods | | | |  |
| Study design | 4 | Present key elements of study design early in the paper | 5, 6 | The study was conducted in a central prison hospital in Jiangsu Province, China. As it mentioned before[10], the individuals who are incarcerated and have been diagnosed with infectious diseases such as HIV, tuberculosis, gonorrhea, syphilis, and other similar conditions in the province are transferred to this hospital for enhanced monitoring and healthcare. |
| Setting | 5 | Describe the setting, locations, and relevant dates, including periods of recruitment, exposure, follow-up, and data collection | 5, 6 | The study was conducted in a central prison hospital in Jiangsu Province, China. As it mentioned before[10], the individuals who are incarcerated and have been diagnosed with infectious diseases such as HIV, tuberculosis, gonorrhea, syphilis, and other similar conditions in the province are transferred to this hospital for enhanced monitoring and healthcare. |
| Participants | 6 | (*a*) *Cohort study*—Give the eligibility criteria, and the sources and methods of selection of participants. Describe methods of follow-up  *Case-control study*—Give the eligibility criteria, and the sources and methods of case ascertainment and control selection. Give the rationale for the choice of cases and controls  *Cross-sectional study*—Give the eligibility criteria, and the sources and methods of selection of participants | 6 | Every individual underwent both QFT-GIT and QFT-Plus tests, and those with active tuberculosis were excluded from our study. PLHIV who have initial *Mycobacterium* tuberculosis infection or receive negative results but pretended to, will be offered preventive treatments. |
|  |  | (*b*) *Cohort study*—For matched studies, give matching criteria and number of exposed and unexposed  *Case-control study*—For matched studies, give matching criteria and the number of controls per case |  |  |
| Variables | 7 | Clearly define all outcomes, exposures, predictors, potential confounders, and effect modifiers. Give diagnostic criteria, if applicable | 7 | Results were considered positive when the IFN-γ concentration in the *Mycobacterium* tuberculosis antigen tube (TB for QFT-GIT and either TB1 or TB2 for QFT-Plus) exceeded the IFN-γ concentration in the nil tube by at least 0.35 IU/ml and was at least 25% of the value in the nil tube. Results were considered indeterminate if the IFN-γ concentration in the nil tube was greater than 8.0 IU/ml, or if the IFN-γ concentration in the mitogen tube was less than 0.5 IU/ml. QFT-GIT test reversion and conversion are defined as changes in the test results from positive to negative and negative to positive, respectively, with a threshold of 0.35 IU/ml. |
| Data sources/ measurement | 8* | For each variable of interest, give sources of data and details of methods of assessment (measurement). Describe comparability of assessment methods if there is more than one group | *6* | This research was conducted as part of the regular health assessments carried out for all provincial prisons. These assessments encompassed standard procedures such as blood tests, biochemical analyses, hepatitis B serology tests, syphilis antibody tests, hepatitis C antibody tests, electrocardiogram evaluations, chest X-ray examinations, CD4 cell counts, and HIV viral load assessments. The majority of individuals closely resembled those described in the published paper, with the exception of prisoners who were either temporarily outside of the prison or newcomers during the specified periods |
| Bias | 9 | Describe any efforts to address potential sources of bias |  | Not applicable |
| Study size | 10 | Explain how the study size was arrived at |  | Not applicable |

Continued on next page

| Quantitative variables | 11 | Explain how quantitative variables were handled in the analyses. If applicable, describe which groupings were chosen and why | 7 | We employed 2x2 contingency tables alongside means accompanied by standard deviations (SD) for the consolidation of continuous and categorical variables. |
| --- | --- | --- | --- | --- |
| Statistical methods | 12 | (*a*) Describe all statistical methods, including those used to control for confounding | 7 | The Fisher exact test or chi-square test was selected for the comparison of the two tests, depending on appropriateness. Quantitative data was compared by Paired-Samples T test. We also conducted a comparison of binary events in QFT-GIT and QFT-Plus using Cohen's kappa (k) coefficient. Kappa coefficients were categorized as follows: poor (k ≤ 0.20), fair (0.20 < k ≤ 0.40), moderate (0.40 < k ≤ 0.60), good (0.60 < k ≤ 0.80), and very good (0.80 < k ≤ 1.00). Bivariate correlation was used to compare quantitative IFN-γ levels between QFT-GIT and QFT-Plus. |
|  |  | (*b*) Describe any methods used to examine subgroups and interactions |  | Not applicable |
|  |  | (*c*) Explain how missing data were addressed |  | Not applicable |
|  |  | (*d*) *Cohort study*—If applicable, explain how loss to follow-up was addressed  *Case-control study*—If applicable, explain how matching of cases and controls was addressed  *Cross-sectional study*—If applicable, describe analytical methods taking account of sampling strategy |  |  |
|  |  | (*e*) Describe any sensitivity analyses |  | Not applicable |
| Results | | | | |
| Participants | 13* | (a) Report numbers of individuals at each stage of study—eg numbers potentially eligible, examined for eligibility, confirmed eligible, included in the study, completing follow-up, and analysed | 8 | A total of 232 PLHIV patients were included in this study, of which 214 (92.2%) had QFT-GIT, EC skin test and TST before. |
|  |  | (b) Give reasons for non-participation at each stage | 8 | A total of 232 PLHIV patients were included in this study, of which 214 (92.2%) had QFT-GIT, EC skin test and TST before. |
|  |  | (c) Consider use of a flow diagram |  | Not applicable |
| Descriptive data | 14* | (a) Give characteristics of study participants (eg demographic, clinical, social) and information on exposures and potential confounders | 8 | Among 232 PLHIV patients, most of them (87.5%) were males. Median age (interquartile range, IQR) was 48 (40-54) years. About a quarter of individuals had a CD4 count of ≥ 500 cells/mm^3^ with the median CD4 (IQR) 369 (265-500) cells/mm^3^. The positive rates of initial QFT-GIT, EC skin test and TST test having before were 25.0%, 14.5% and 15.4%, respectively. A total of 47 individuals (74.6%) underwent the 3HP therapy, while 16 individuals (25.4%) received the *Mycobacterium* Vaccae treatment. |
|  |  | (b) Indicate number of participants with missing data for each variable of interest |  | Not applicable |
|  |  | (c) *Cohort study*—Summarise follow-up time (eg, average and total amount) |  |  |
| Outcome data | 15* | *Cohort study*—Report numbers of outcome events or summary measures over time |  |  |
|  |  | *Case-control study—*Report numbers in each exposure category, or summary measures of exposure |  |  |
|  |  | *Cross-sectional study—*Report numbers of outcome events or summary measures | *8* | In total, 57 patients (24.6%) were identified with *Mycobacterium* tuberculosis infection based on the outcomes of the QFT-GIT test, while 56 patients (24.1%) were diagnosed for *Mycobacterium* tuberculosis infection using the QFT-Plus. |
| Main results | 16 | (*a*) Give unadjusted estimates and, if applicable, confounder-adjusted estimates and their precision (eg, 95% confidence interval). Make clear which confounders were adjusted for and why they were included |  | Not applicable |
|  |  | (*b*) Report category boundaries when continuous variables were categorized |  | Not applicable |
|  |  | (*c*) If relevant, consider translating estimates of relative risk into absolute risk for a meaningful time period |  | Not applicable |

Continued on next page

| Other analyses | 17 | Report other analyses done—eg analyses of subgroups and interactions, and sensitivity analyses |  | Not applicable |
| --- | --- | --- | --- | --- |
| Discussion | | | | |
| Key results | 18 | Summarise key results with reference to study objectives | 11 | In this research, we assessed the diagnostic potential of both QFT-GIT and QFT-Plus in detecting *Mycobacterium* tuberculosis infection across PLHIV in a prison hospital. There was no notable distinction observed in FN-γ levels when comparing the QFT-GIT and QFT-Plus assays and the effectiveness of QFT-GIT and QFT-Plus in identifying *Mycobacterium* tuberculosis infection among PLHIV with relatively higher CD4 count levels was found to be comparable, and the correspondence between these two tests within the PLHIV group demonstrated a strong level of concurrence, exceeding 90%. Furthermore, our investigation revealed that among PLHIV regardless of whether it's the chemotherapy regimen or the immunotherapy regimen, preventive treatment for *Mycobacterium* tuberculosis infection would lead to a reduction in IFN-γ levels. |
| Limitations | 19 | Discuss limitations of the study, taking into account sources of potential bias or imprecision. Discuss both direction and magnitude of any potential bias | 14 | Several limitations are present in our study. Firstly, we did not conduct the T-SPOT.TB or TST assays concurrently, which could have served as a reference standard for assessing the precision of the QFT-GIT and QFT-Plus assays. Secondly, we were unable to differentiate whether the decline in IFN-γ levels resulted from preventive treatment, natural clearance, or within-subject variability in T-cell responses, given the absence of a parallel control group. |
| Interpretation | 20 | Give a cautious overall interpretation of results considering objectives, limitations, multiplicity of analyses, results from similar studies, and other relevant evidence | 11-14 |  |
| Generalisability | 21 | Discuss the generalisability (external validity) of the study results |  | Not applicable |
| Other information | |  | | |
| Funding | 22 | Give the source of funding and the role of the funders for the present study and, if applicable, for the original study on which the present article is based | 17 | This study was funded by the work was supported by “Research and Pre-research Fund of the Second Affiliated Hospital of Soochow University(SDFEYJC2326)”, “Jiangsu Provincial Medical Key Discipline” (ZDXK202250), “Jiangsu Provincial Association for Science and Technology Youth Science and Technology Talent Support Project”(JSTJ-2023-WJ007) and “Jiangsu Province Preventive Medicine Research Topic Surface Project”(Ym2023039). |

*Give information separately for cases and controls in case-control studies and, if applicable, for exposed and unexposed groups in cohort and cross-sectional studies.

**Note:** An Explanation and Elaboration article discusses each checklist item and gives methodological background and published examples of transparent reporting. The STROBE checklist is best used in conjunction with this article (freely available on the Web sites of PLoS Medicine at http://www.plosmedicine.org/, Annals of Internal Medicine at http://www.annals.org/, and Epidemiology at http://www.epidem.com/). Information on the STROBE Initiative is available at www.strobe-statement.org.
